# Magnetic Resonance Elastography of Invasive Breast Cancer: Evaluating Prognostic Factors and Treatment Response

**DOI:** 10.3390/tomography11020018

**Published:** 2025-02-14

**Authors:** Jin Joo Kim, Jin You Kim, Yeon Joo Jeong, Suk Kim, In Sook Lee, Nam Kyung Lee, Taewoo Kang, Heeseung Park, Seokwon Lee

**Affiliations:** 1 Department of Radiology, Medical Research Institute, Pusan National University Hospital, Pusan National University School of Medicine, Busan 49241, Republic of Korea; wwn35@hanmail.net (J.J.K.);; 2 Department of Radiology, Pusan National University Yangsan Hospital, Pusan National University School of Medicine, Yangsan-si 50612, Republic of Korea; 3 Busan Cancer Center and Biomedical Research Institute, Department of Surgery, Pusan National University Hospital, Pusan National University School of Medicine, Busan 49241, Republic of Korea; 4 Department of Surgery, Pusan National University Hospital, Pusan National University School of Medicine, Busan 49241, Republic of Korea

**Keywords:** breast, magnetic resonance imaging, elasticity imaging techniques, breast neoplasms, prognosis

## Abstract

**Objectives:** To assess the elasticity values in breast tissues using magnetic resonance elastography (MRE) and examine the association between elasticity values of invasive breast cancer with prognostic factors and the pathologic response to neoadjuvant systemic therapy (NST). **Methods:** A total of 57 patients (mean age, 54.1 years) with invasive breast cancers larger than 2 cm in diameter on ultrasound were prospectively enrolled. The elasticity values (mean, minimum, and maximum) of invasive breast cancers, normal fibroglandular tissues, and normal fat tissues were measured via MRE using a commercially available acoustic driver and compared. Elasticity values of breast cancers were compared according to prognostic factors and pathologic responses in patients who received NST before surgery. Receiver operating curve analysis was performed to evaluate the predictive efficacy of elasticity values in terms of pathological response. **Results:** Among the 57 patients, the mean elasticity value of invasive breast cancers was significantly higher than that of normal fibroglandular tissue and normal fat tissue (7.90 ± 5.80 kPa vs. 2.54 ± 0.80 kPa vs. 1.32 ± 0.33 kPa, all *p*s < 0.001). Invasive breast cancers with a large diameter (>4 cm) exhibited significantly higher mean elasticity values relative to tumors with a small diameter (≤4 cm) (11.65 ± 7.22 kPa vs. 5.87 ± 3.58 kPa, *p* = 0.002). Among 24 patients who received NST, mean, minimum, and maximum elasticity values significantly differed between the pathologic complete response (pCR) and non-pCR groups (all *p*s < 0.05). For the mean elasticity value, the area under the curve value for distinguishing pCR and non-pCR groups was 0.880 (95% confidence interval, 0.682, 0.976; *p* < 0.001). **Conclusions:** The elasticity values of invasive breast cancers measured via breast MRE showed a positive correlation with tumor size and showed potential in predicting the therapeutic response in patients receiving NST.

## 1. Introduction

Tumor stiffness is an important biomechanical property influenced by the complex interactions between cells and the extracellular matrix, and higher stiffness may contribute to tumor progression [1]. Increased tissue hardness is an obvious mechanical abnormality in many malignant tumors, including breast cancers [2]. Ultrasound elastography is a commonly utilized method for assessing tumor stiffness noninvasively. Shear wave elastography (SWE) is one of the most frequently used ultrasound techniques, and it allows quantitative measurement of the tumor stiffness through a focused ultrasound beam inducing mechanical vibrations [3]. However, ultrasound elastography has certain limitations, including operator dependency, limited depth penetration, and suboptimal spatial resolution [4].

Magnetic resonance elastography (MRE) is an advanced, noninvasive magnetic resonance imaging (MRI) technique that provides quantitative viscoelastic properties of tissues [5]. Unlike ultrasound, MRE can measure motions in arbitrary directions with equal sensitivity and high accuracy [6]. MRE is increasingly employed in clinical practice to assess hepatic fibrosis; it exhibits robust diagnostic performance when evaluating hepatic malignancy [7]. In the context of breast imaging, MRE remains in a state of evolution, and research is ongoing in the experimental phase to utilize it for evaluating breast tissue [5]. Previous studies have shown the potential for MRE combined with contrast-enhanced MRI to improve diagnostic accuracy in differentiating benign and malignant breast lesions [8,9,10]. However, the result regarding the correlation between MRE-derived elasticity values and prognostic features of breast cancer is limited.

Several studies have demonstrated associations between tumor stiffness measured by SWE and prognostic factors [11,12,13,14,15,16]. These findings suggest that traditional poor prognostic factors of breast cancer, including larger tumor size, higher histological and nuclear grades, presence of axillary lymph node metastasis, and lymphovascular invasion, correlate with higher elasticity values determined by SWE [11,12,13]. In terms of tumor subtypes, more aggressive subtypes demonstrate higher stiffness in comparison to tumors that are estrogen receptor (ER)-positive [15,16]. Furthermore, several studies have explored the potential of stiffness measurements obtained via SWE in terms of predicting the efficacy of neoadjuvant systemic therapy (NST) [17,18,19,20]. As higher tumor elasticity values measured via SWE were significantly correlated with worse prognostic factors, we hypothesized that tumor elasticity values assessed by MRE might also provide additional information on the prognosis and treatment response of breast cancers.

Therefore, the two aims of this prospective study were:

(1) to determine whether the elasticity values of invasive breast cancer were correlated with prognostic factors;
(2) to evaluate whether elasticity values can predict the response to NST in patients with invasive breast cancer.

## 2. Materials and Methods

### 2.1. Patients

This prospective single-institution study was approved by our Institutional Review Board (IRB No. 2204-024-113). From July 2022 to April 2023, we recruited patients with core needle biopsy-proven invasive breast cancers larger than 2 cm on diagnostic ultrasound. Written informed consent was obtained from all participants. Because of this study design, where we positioned a passive driver on the patient’s back to transmit waves to the breast, patients with breast cancers larger than 2 cm on diagnostic ultrasound were included in this analysis. In total, 63 patients with invasive breast cancers were recruited. Among them, five did not undergo either surgery or NST, and one patient was diagnosed with diffuse large B-cell lymphoma after repeated core needle biopsies. Finally, 57 patients with invasive breast cancers larger than 2 cm in diameter from diagnostic ultrasound were enrolled. For 14 women with multifocal or multicentric breast tumors, only the largest tumors were included in the analysis.

Of the 57 patients, 24 received NST prior to breast surgery; 9 patients were prescribed four cycles of doxorubicin (Adriamycin) and cyclophosphamide (Cytoxan) followed by four cycles of docetaxel. A total of 15 patients with human epidermal growth factor receptor 2 (HER2)-positive tumors received six cycles of docetaxel/carboplatin/trastuzumab/pertuzumab regimen.

Thirty-three patients underwent breast-conserving surgery *(n* = 15) or mastectomy (*n* = 18) without receiving neoadjuvant therapy. In those patients, the median interval between MRI acquisition and surgery was 10 days (range, 3–19 days).

### 2.2. MRI Acquisition

All breast MRI examinations were performed using a 3-T system (Signa Premier; General Electric Healthcare, Milwaukee, WI, USA) with a dedicated 8-channel breast coil (General Electric Healthcare); each patient was imaged in the prone position. The imaging protocol started with a localizing sequence followed by axial fat-suppressed T2-weighted fast spin-echo imaging (TR/TE = 10,454/105.35 ms; matrix size = 1024 × 1024; field of view (FOV) = 350 × 350 mm^2^; slice thickness = 3.0 mm). After the acquisition of T2-weighted images, MRE was performed after applying mechanical vibrations supplied by an active pneumatic driver system (General Electric Healthcare, Milwaukee, WI, USA) at 60 Hz frequency. These vibrations were located outside the scanning room and delivered the waves to the center of the patient’s back via a plastic tube that terminated in a passive rigid round driver (General Electric Healthcare, Milwaukee, WI, USA), which facilitated the transmission of the waves into the breasts. The passive driver was fastened to the center of the patient’s back using an elastic belt. MRE was acquired using a gradient-echo echo-planar imaging pulse sequence (oblique axial direction, TR/TE = 2100.67/65.9 ms; matrix size = 256 × 256; FOV = 350 × 350 mm^2^; slice thickness 5 mm; parallel imaging acceleration factor of 2). The total acquisition time of the MRE was 4 min 48 s.

Dynamic contrast-enhanced (DCE)-MRI was conducted utilizing a fat-suppressed T1-weighted volume imaging for breast assessment (TR/TE = 4.17/1.7 ms; matrix size = 512 × 512; flip angle = 12°; FOV = 370 × 370 mm^2^; slice thickness, 1.79 mm, and no gap). The imaging protocol comprised one pre-contrast image acquisition, followed by five post-contrast image acquisitions. For contrast administration, an intravenous bolus of 0.1 mmol/kg gadobutrol (Gadovist; Bayer Schering Pharma, Berlin, Germany) was infused at a rate of 1.5 mL/s via a power injector, accompanied by an immediate 20-mL saline flush at the same rate. Subsequently, post-contrast images were obtained at 60, 140, 220, 300, and 380 s after contrast injection.

### 2.3. Image Analyses

All MR images were reviewed on the monitors of picture archiving and communication system workstations (Infinite^®^; Marotech, Seoul, Republic of Korea) by a radiologist (J.J.K. with 4 years of experience in breast MRI). The reviewer was informed of the breast cancer diagnosis but was blinded to the clinico-histopathological data. Maximal tumor diameters were determined by measuring the single largest diameters in the first phase of DCE MR images. When multiple tumors were present, only the largest tumor was included in the analysis.

**Figure 1:**
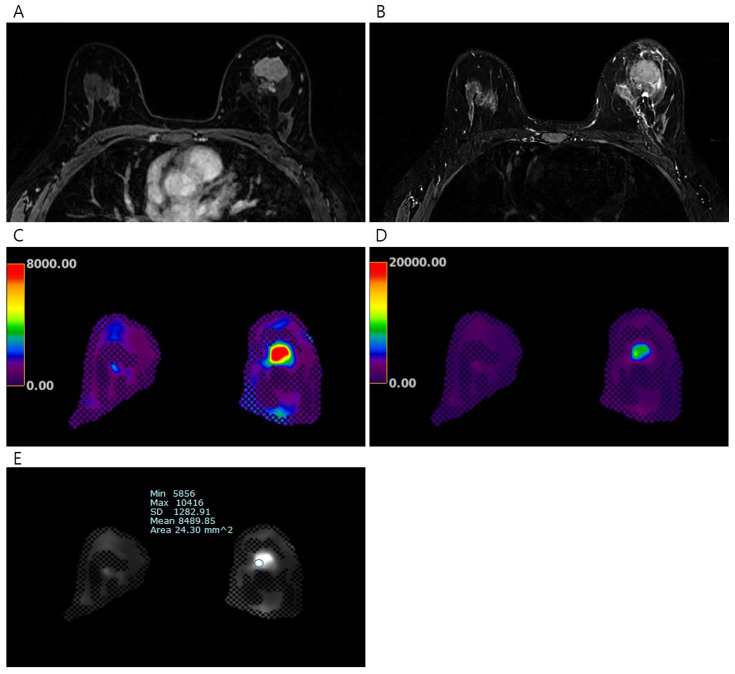
Pretreatment MRI of the breasts in 45 years-old-woman with invasive breast cancer. (**A**) Early phase of dynamic contrast-enhanced T1-weighted image shows an irregular heterogeneous enhancing mass in the left breast, (**B**) T2-weighted image shows irregular hyperintense mass, (**C**) the processed color map of elastography with a range of 0–8 kPa shows a mass with red color, and (**D**) the process color map of elastography with a range of 0–20 kPa shows a mass with green color. The elasticity values were measured via (**E**) elastography by drawing region of interest. The mean elasticity value of the breast cancer was 8.49 kPa. After completion of neoadjuvant chemotherapy, no residual cancer was found on surgical histopathology (ypT0N0).

**Figure 2:**
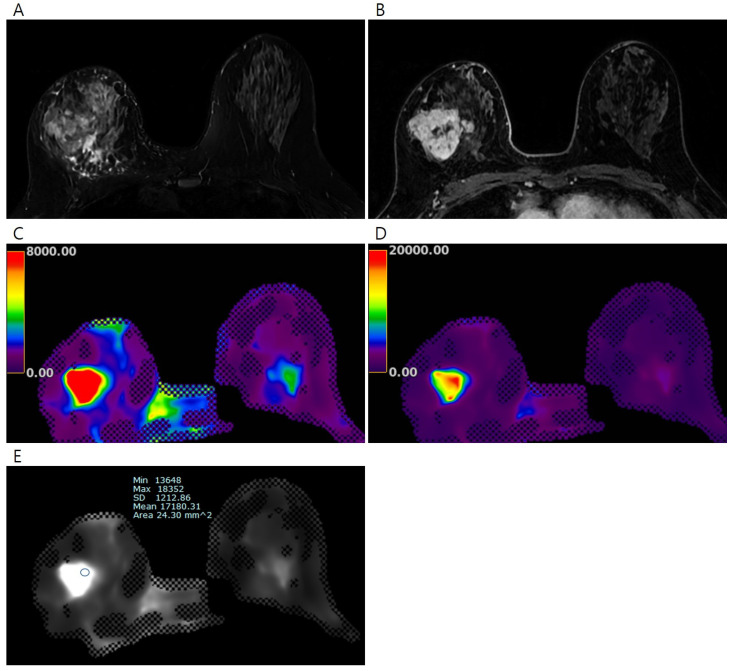
Pretreatment MRI of the breasts in 45 years-old-woman with invasive breast cancer. (**A**) Early phase of the dynamic contrast-enhanced T1-weighted image shows an irregular heterogeneous enhancing mass in the right breast, (**B**) T2-weighted image shows an irregular hyperintense mass in the right breast, the processed color maps of elastography with a range of (**C**) 0–8 kPa and (**D**) 0–20 kPa show a stiff mass with red color. The elasticity values were measured via (**E**) elastography by drawing region of interest. The mean elasticity value of the breast cancer was 17.18 kPa. After completion of neoadjuvant chemotherapy, a 5.2 cm-sized residual mixed mucinous and invasive ductal carcinoma was found on surgical histopathology (ypT3N1).

The color maps of the elastography were processed over two ranges (0–8 kPa and 0–20 kPa); the colors ranged from dark purple (lowest stiffness) to red (highest stiffness). To measure the elasticity value of the invasive breast cancer, the reviewers identified the stiffest lesions using both the DCE-MRI data and the processed color maps of elastography (Figure 1 and Figure 2). Subsequently, a fixed circular region of interest (ROI) with an area of 24.30 mm^2^ was placed on each gray-scale elastography image over the stiffest part of the lesion. ROIs with the same size were also placed on normal fibroglandular and normal fat tissues of the contralateral breast at nipple level, guided by the DCE-MRI. Mean, minimum, and maximum elasticity values were recorded for invasive breast cancers, normal fibroglandular tissues, and normal fat tissues. To assess the reproducibility of elasticity value measurements, second measurements on the invasive breast cancer were independently conducted by two radiologists (J.J.K. and J.Y.K., 4 years and 10 years of experience in breast MRI, respectively) using the same method as the first measurement.

### 2.4. Clinico-Histopathological Analysis

All clinico-histopathological information was obtained from electronic medical records. For the 33 patients who underwent surgery without receiving NST, information about their histological type and histological grade of the tumors was collected from pathology reports regarding surgical specimens. Immunohistochemical findings, including ER status, progesterone receptor (PR) status, and HER2 and Ki-67 statuses, were extracted from pathology reports concerning surgical specimens of patients who underwent surgery without receiving NST. For the 24 patients who received NST before surgery, information about their histological type, histological grade, and immunohistochemical findings of the tumors was collected from pathology reports of core needle biopsies performed prior to the initiation of NST. The Allred score was used to determine the levels of ER and PR expression [21]. The HER2 score was stratified into 0, 1+, 2+, or 3+. Tumors with HER2 scores 3+ and/or HER2 gene amplification, confirmed via fluorescence in situ hybridization (FISH), were regarded as HER2-positive [22]. A Ki-67 nuclear staining index ≥ 20% was considered indicative of high-level Ki-67 expression [23]. Based on the immunohistochemical or FISH results for ER, PR, HER2, and Ki-67 expression, tumors were classified into the following three subtypes: ER-positive (ER+/HER2−, PR may be positive or negative), HER2-positive (HER2+, ER, and PR may be positive or negative), and triple-negative (ER/PR/HER2−).

All 24 patients who received NST underwent breast-conserving surgery or mastectomy after NST completion. A pathological complete response (pCR) was defined as the absence of invasive tumor cells in the excised specimen (stage ypT0). Pathology assessments of biopsy and surgical tumor specimens were performed by a breast pathologist.

### 2.5. Statistical Analysis

The elasticity values of normal fat tissue, normal fibroglandular tissue, and invasive breast cancer were compared using two-sample *t*-tests. The Kruskal–Wallis test, followed by the Mann–Whitney U test with Bonferroni correction for multiple comparisons, was used to evaluate the differences in elasticity values according to clinico-histopathological features and clinico-histopathological characteristics according to pathological responses. When comparing elasticity values with pathological responses, the Student’s *t*-test or the Wilcoxon rank-sum test was performed if the Kolmogorov–Smirnov test revealed a normal distribution. The predictive capacities of elasticity values in terms of pathological response were evaluated by generating receiver operating characteristic curves (ROCs) and determining areas under the curves (AUCs). The intraclass correlation coefficients were derived to assess intraobserver and interobserver reproducibility.

Statistical analyses were performed using IBM SPSS ver. 27.0.0 (IBM Co., Armonk, NY, USA) and MedCalc ver. 22.021 (MedCalc Software, Mariakerke, Belgium). Differences were considered statistically significant at *p*-values < 0.05.

## 3. Results

### 3.1. Baseline Characteristics

Patient and tumor characteristics are summarized in Table 1. In total, 57 women (mean age, 54.1 years; range, 27–76 years) were included. Among them, 18 (32%) were asymptomatic and 39 (68%) had symptoms; 35 (61%) had lumps, 3 (5%) had breast pain, and 1 (2%) had nipple retraction. The majority of invasive cancers were invasive ductal carcinomas (50 of 57 [88%]) followed by invasive lobular carcinoma (3 of 57 [5%]), mucinous carcinomas (3 of 57 [5%]), and metaplastic carcinoma (1 of 57 [2%]). Of the 57 invasive breast cancers, 41 (72%) were histological grade 2, and 16 (28%) were histological grade 3. Regarding tumor subtypes, 34 (60%) were ER-positive, 13 (23%) were HER2-positive, and 10 (17%) were triple-negative breast cancers. The median interval between core needle biopsy and MRI was 21 days (range, 14–35 days).

### 3.2. Elasticity Values of Breast Tissue

Table 2 lists the mean, minimum, and maximum elasticity values of normal fat tissues, normal fibroglandular tissues, and cancer tissues for all patients. The mean elasticity value in cancer was 7.90 ± 5.80 kPa; the mean elasticity values of normal fat and normal fibroglandular tissues were 1.32 ± 0.33 kPa and 2.54 ± 0.80 kPa, respectively. Statistically significant differences were observed in the mean, minimum, and maximum elasticity values between invasive cancer and normal fibroglandular or normal fat tissues (all *p*s < 0.001).

The intraclass correlation coefficients (ICCs) for interobserver agreements of the mean, minimum, and maximum elasticity values of invasive breast cancer were 0.908 (95% confidence interval [CI] 0.844, 0.946), 0.664 (95% CI 0.429, 0.802), and 0.787 (95% CI 0.639, 0.875), respectively. For intraobserver measurement reliability, the ICCs of the mean, minimum, and maximum elasticity values of invasive breast cancers were 0.947 (95% CI: 0.910, 0.969), 0.744 (95% CI: 0.580, 0.843), and 0.810 (95% CI: 0.797, 0.840), respectively.

### 3.3. Mean Elasticity Values of Invasive Breast Cancers According to Clinico-Histopathological Features

Of 57 invasive breast cancers, 37 (65%) had tumors with smaller size (maximal tumor diameter ≤ 4 cm), and 20 (35%) had tumors with larger size (maximal tumor diameter > 4 cm). Tumors with larger size exhibited higher mean elasticity values than tumors with smaller size (11.65 ± 7.21 kPa vs. 5.87 ± 3.58 kPa, *p* = 0.002). The mean elasticity value did not significantly differ according to age, menopausal status, histological grade, histological type, or tumor subtype (Table 3).

### 3.4. Clinico-Histopathological Characteristics and Elasticity Values According to Response to Neoadjuvant Systemic Therapy

**Figure 3:**
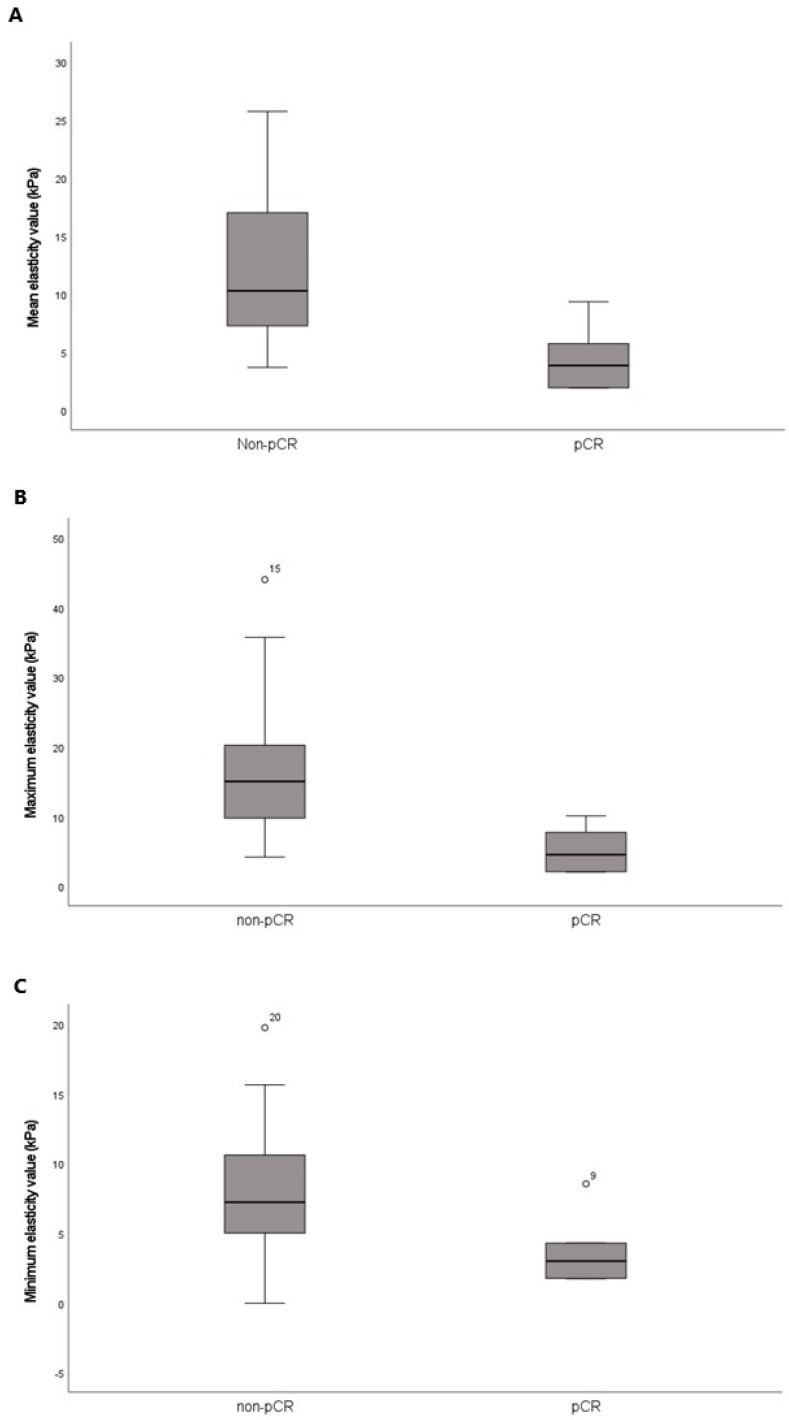
Box-and-whisker plots of the pretreatment (**A**) mean, (**B**) maximum, and (**C**) minimum elasticity values according to the response to neoadjuvant systemic therapy. The horizontal line within each box is the median; the top and bottom edges of each box are the 25% and 75% percentiles of elasticity values, respectively.

Of the 24 patients who received NST before surgery, 6 (25%) achieved pCR, while 18 (75%) did not (Table 4). The pCR group exhibited a smaller maximal tumor diameter on pretreatment MRI than the non-pCR group (2.52 ± 1.11 cm vs. 5.19 ± 2.70 cm, *p* = 0.007). Among the categorical variables, HER2 status significantly differed between the pCR and non-pCR groups (*p* < 0.001). However, age, menopausal status, histological grade, histological type, tumor subtype, ER, PR, and Ki-67 status were not different between the two groups. Mean, minimum, and maximum elasticity values were significantly lower in the pCR group relative to the non-pCR group (4.45 ± 2.81 kPa vs. 12.20 ± 6.71 kPa, *p* < 0.001; 3.72 ± 2.55 kPa vs. 8.11 ± 4.86 kPa, *p* = 0.047; 5.19 ± 3.22 kPa vs. 16.97 ± 10.79 kPa, *p* = 0.016, respectively) (Table 5 and Figure 3).

### 3.5. ROC Analysis

**Figure 4:**
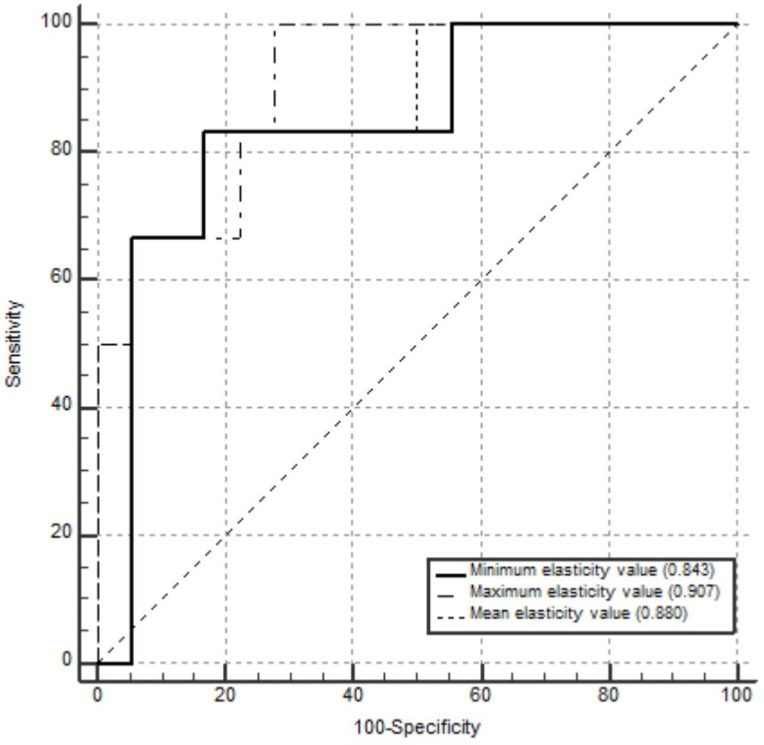
Receiver operating characteristic curves for the mean, maximum, and minimum elasticity values used as predictors of pathological complete response in 24 patients who received neoadjuvant systemic therapy. The areas under the curves for pathological complete response prediction were 0.880 (95% confidence interval [CI] 0.682, 0.976) for mean elasticity value, 0.907 (95% CI 0.718, 0.987) for maximum elasticity value, and 0.843 (95% CI 0.637, 0.958) for minimum elasticity value.

The AUCs for pCR prediction were 0.880 (95% CI 0.682, 0.976) for the mean elasticity value, 0.843 (95% CI 0.637, 0.958) for the minimum elasticity value, and 0.907 (95% CI 0.718, 0.987) for the maximum elasticity value. The results of the ROC analyses are shown in Figure 4.

## 4. Discussion

To the best of our knowledge, this is the first clinical trial that assessed the utility of a breast MRE technique using a commercially available acoustic driver (GE Healthcare) designed for the evaluation of hepatic fibrosis. We assessed the elasticity values of invasive breast cancers, normal fibroglandular tissues, and normal fat tissues and observed significant differences in elasticity values between invasive breast cancer and normal fibroglandular or fat tissues. We also evaluated associations between the elasticity values and the clinico-histopathological factors of invasive breast cancers and whether the elasticity values predicted pCR in patients who received NST before surgery. The results showed that tumor size emerged as the sole factor associated with a significant difference according to the elasticity values. Among patients receiving NST, those who achieved pCR exhibited significantly lower mean, minimum, and maximum elasticity values than those who did not achieve pCR. These findings suggest that elasticity values measured via MRE can be used to aid the diagnosis of invasive breast cancer and may be a candidate imaging biomarker to predict pathological responses in patients who receive NST.

Elasticity, an intrinsic mechanical property of any tissue, reflects how the tissue deforms under mechanical pressure. MRE can measure this elastic shear property by direct visualization of acoustic waves within the tissue [24]. To obtain breast MRE images, adequate shear waves must be delivered to breast tissues [5]. To date, two methods have been employed for this purpose, one of which uses an active driver in direct contact with the breast tissue; however, this driver may exert a compressive effect [8,9,25,26,27]. The other method utilizes a non-compressive passive driver positioned between the sternum and the radiofrequency coil [10,24]. Thus far, neither approach has been standardized. Here, we used a commercially available driver originally designed for the assessment of hepatic fibrosis to obtain breast MRE images. Our results revealed that invasive breast cancers were significantly stiffer than normal breast tissues (*p* < 0.001). Similarly, in a preliminary study from 2002, McKnight et al. reported that shear stiffness was significantly higher in tumors than in adipose breast tissues among six patients with invasive breast cancer (*p* < 0.05) [8]. They noted that the mean shear stiffness was 418% higher in breast cancers than in the surrounding breast tissue [8]. Lorenzen et al. concluded that malignant lesions exhibited a higher median elasticity of 15.9 kPa compared with benign lesions (median 7 kPa [*p* = 0.0012]) [28]. Xydeas et al. also reported the malignant lesions were stiffer than benign breast disease or normal breast tissue [29]. The consistent trend of differing elasticity values between malignant tumors and surrounding breast tissues underscores the potential of MRE as an imaging technique to differentiate malignant breast tumors from benign breast tissues. Notably, similar results are achieved even with the use of a passive driver originally designed for liver assessments, suggesting that the breast MRE technique using a commercially available driver designed for assessing hepatic fibrosis can be also feasible for breast evaluation.

SWE is a widely utilized US-based imagining technique, which allows quantitative assessment of tissue stiffness. In SWE, a focused US acoustic wave from the probe generates a mechanical vibration, and the speed of the wave within the tissue is measured to assess tissue stiffness [3]. Because the size of the probe is generally limited to 5 cm, very large tumors may extend beyond the maximum SWE overlay or even beyond the FOV. In such cases, SWE may pose challenges, as it might not image the stiffest portion of the mass or might not perceive the surrounding tissue [11]. MRE provides viscoelastic information through direct MR visualization of acoustic waves by characterizing the tissue response to the stress [24]. As such, MRE enables an objective and comprehensive analysis of the breasts, especially in patients with large tumors. In this study, large tumors (maximal diameter larger than 4 cm) demonstrated a significantly higher mean elasticity value than that of small tumors (maximal diameter smaller than 4 cm). Previous studies using SWE have reported an association between tumor size and stiffness [15,30,31]. Specifically, tumors larger than 2 cm were found to have higher stiffness compared to those smaller than 1 cm [15,31]. However, tumors larger than 5 cm were excluded from SWE studies due to limitations regarding depth penetration and spatial resolution [15], while tumors smaller than 2 cm were excluded from this study. Therefore, the previous results of SWE studies are not directly comparable to the findings of this study, but the tendency for larger tumors to exhibit higher stiffness is consistent. This result may be explained by the extracellular matrix, which becomes denser as the size of the breast cancer increases, contributing to greater tumor stiffness [32]. A denser extracellular matrix promotes cancer cell proliferation and thus is associated with a poor prognosis in patients with breast cancer [33]. Unlike the present study, previous SWE studies have found positive correlations between tumor stiffness and prognostic factors, such as higher histological and nuclear grades, the presence of axillary lymph node metastasis, and lymphovascular invasion [11,12,13]. The small sample size and disparity in the study population might contribute to the different results.

NST is increasingly used to downstage locally advanced breast cancers, thereby potentially facilitating breast-conserving surgery. Achievement of pCR is considered the optimal outcome of NST; this outcome is associated with an improvement in overall and disease-free survival. Analyses using SWE have consistently revealed that a low elasticity value within invasive breast cancer is associated with a favorable response to NST [20,34,35]. In this study, utilizing MRE to measure elasticity values, we obtained similar results, that is, patients achieving pCR exhibited significantly lower elasticity values compared with those who did not achieve pCR. In a recent animal study that combined MRE with computational histopathology, Li et al. demonstrated that the quantification of elasticity serves as a sensitive imaging biomarker of tumor collagen deposition and enzymatic degradation of the collagen network [36]. Collagen significantly increases tumor stiffness, and it plays a vital role in the progression of breast cancer and resistance to therapy by high interstitial fluid pressure, which eventually limits the delivery of chemotherapeutic drugs [37,38]. Thus, by providing in vivo elasticity information, MRE may provide additional biomechanical information in the context of treatment response. However, considering the small sample size of our study, it is challenging to disregard the existence of an interaction between tumor size and elasticity value in terms of the pathological response to NST.

Our study had several limitations. First, this was a single-center prospective study that included a small sample size with selection bias. Additionally, the relatively small number of patients who received NST may have limited the statistical power of the analysis. Second, we did not perform mid- or post-treatment MRE on patients receiving NST; such an evaluation might have provided more comprehensive information about tumor responses to NST. Third, we used information regarding tumor histological types, histological grades, and immunochemical profiles derived from two different types of specimens: surgical specimens from patients who underwent surgery without receiving NST and core needle biopsies from patients who received NST before surgery. Because the latter specimens are derived from only small portions of tumors, the corresponding whole-tumor characteristics may differ from those of biopsy samples. Additionally, prognostic factors regarding the presence of axillary lymph node metastasis and lymphovascular invasion could not be evaluated because information about these factors can only be obtained from a surgical specimen. Fourth, each elasticity value was assessed using only a small ROI in a representative slice; this value may not reflect whole-tumor characteristics. Recently, a three-dimensional MRE sequence has been developed, which could yield volumetric information about the tumor [24]. Further analysis using the three-dimensional MRE protocol might facilitate a comprehensive understanding of tumor biology. Finally, in this study, a passive driver initially developed to assess hepatic fibrosis was placed on the back of each patient; this driver may not have provided optimal delivery of acoustic waves to breast tissues. Additional large-scale multi-institutional studies are needed for validation and generalization of the MRE results.

## 5. Conclusions

Large tumors (maximal diameter larger than 4 cm) exhibited higher elasticity values compared with small tumors (maximal diameter less than 4 cm). In patients who received NST, those achieving pCR exhibited significantly lower mean, minimum, and maximum elasticity values compared with those who did not achieve pCR. Our results provide preliminary evidence that elasticity values measured via MRE show potential in predicting pathological responses in breast cancer patients receiving NST before surgery. However, additional prospective studies with large sample sizes are required to elucidate the clinical utility of breast MRE.

## Figures and Tables

**Table 1:** Patient and tumor characteristics.

Variables	All (*n* = 57)
Mean age (years) ^a^	54.05 ± 11.91
Tumor size on pretreatment MRI (cm) ^a,b^	3.96 ± 2.34
Symptoms	
Asymptomatic	18 (32)
Lump	35 (61)
Pain	3 (5)
Nipple retraction	1 (2)
Histological type	
Ductal	50 (88)
Lobular	3 (5)
Mucinous	3 (5)
Metaplastic	1 (2)
Histological grade	
2	41 (72)
3	16 (28)
Estrogen receptor status	
Positive	40 (70)
Negative	17 (30)
Progesterone receptor status	
Positive	29 (51)
Negative	28 (49)
HER2 status	
Positive	13 (23)
Negative	44 (77)
Ki-67 status	
High (>20%)	44 (77)
Low (≤20%)	13 (23)
Tumor subtypes	
ER-positive	34 (60)
HER2-positive	13 (23)
Triple-negative	10 (17)

Note. Unless otherwise stated, data are presented as patient numbers with percentages shown in parentheses. Histological features were determined using specimens obtained during surgery or via core biopsy before chemotherapy. ^a^ Data are presented as means ± standard deviations. ^b^ Tumor size represented maximal tumor diameter measured on DCE MR images. HER2 human epidermal growth factor receptor 2.

**Table 2:** Elasticity values of normal fat tissues, normal fibroglandular tissues, and cancers in all patients (*n* = 57).

	Normal Fat Tissue	Normal Fibroglandular Tissue	Cancer	*p*-Value ^a^	*p*-Value ^b^
Mean elasticity value (kPa)	1.32 ± 0.33	2.54 ± 0.80	7.90 ± 5.80	<0.001	<0.001
Maximum elasticity value (kPa)	1.37 ± 0.34	2.70 ± 0.86	11.79 ± 11.52	<0.001	<0.001
Minimum elasticity value (kPa)	1.26 ± 0.33	2.35 ± 0.73	5.28 ± 3.87	<0.001	<0.001

Note. Data are presented as means ± standard errors. ^a^ *p*-values represents the comparison between cancer and normal fat tissues. ^b^ *p*-values represent the comparison between cancer and normal fibroglandular tissue.

**Table 3:** Relationships between mean elasticity values and the clinico-histopathological features of 57 invasive breast cancers.

Variables	*n*	Mean Elasticity Value (kPa) ^a^	*p*-Value
Age at diagnosis (years)			0.213
≤50	20 (35)	8.83 ± 5.65	
>50	37 (65)	7.40 ± 5.89	
Menopausal status			0.226
Premenopausal	26 (46)	9.34 ± 7.04	
Postmenopausal	31 (54)	6.69 ± 4.26	
Tumor size on pretreatment MRI (cm) ^b^			0.002
≤4	37 (65)	5.87 ± 3.58	
>4	20 (35)	11.65 ± 7.22	
Histological grade			0.338
1	0 (0)		
2	41 (72)	7.70 ± 6.24	
3	16 (28)	8.40 ± 4.65	
Histological type			0.309
Ductal	50 (88)	7.98 ± 5.79	
Lobular	3 (5)	4.46 ± 2.82	
Mucinous	3 (5)	5.91 ± 3.79	
Metaplastic	1 (2)	20.06	
Tumor subtypes			0.183
ER-positive	34 (60)	7.54 ± 5.42	
HER2-positive	13 (23)	7.06 ± 6.69	
Triple-negative	10 (17)	10.17 ± 5.87	

Note. Data are presented as numbers of patients with percentages shown in parentheses unless otherwise indicated. Histological features were determined for specimens obtained during surgery or via core biopsy before chemotherapy. ^a^ Data are presented as means ± standard errors. ^b^ The largest tumor diameter was measured via pretreatment DCE MR images. HER2 human epidermal growth factor receptor 2.

**Table 4:** Clinico-histopathological characteristics of breast cancer patients according to the response to neoadjuvant systemic therapy (*n* = 24).

Variables	Non-Pathological Complete Response (*n* = 18)	Pathological Complete Response (*n* = 6)	*p*-Value
Tumor size on pretreatment MRI (cm) ^a^	5.19 ± 2.70	2.52 ± 1.11	0.007
Age at diagnosis (years)			0.446
≤50	6 (33)	1 (17)	
>50	12 (67)	5 (83)	
Menopausal status			0.752
Premenopausal	8 (44)	2 (33)	
Postmenopausal	10 (56)	4 (67)	
Histological grade			0.446
1	0 (0)	0 (0)	
2	12 (67)	5 (83)	
3	6 (33)	1 (17)	
Histological type			0.564
Ductal	17 (94)	6 (100)	
Metaplastic	1 (6)	0 (0)	
Estrogen receptor status			0.487
Positive	9 (50)	4 (67)	
Negative	9 (50)	2 (33)	
Progesterone receptor status			0.216
Positive	14 (78)	6 (100)	
Negative	4 (22)	0 (0)	
HER2 status			<0.001
Positive	3 (17)	6 (100)	
Negative	15 (183)	0 (0)	
Ki-67 status			0.233
>20%	8 (44)	1 (17)	
≤20%	10 (56)	5 (83)	
Tumor subtypes			0.871
ER-positive	7 (39)	0 (0)	
HER2-positive	3 (17)	6 (100)	
Triple-negative	8 (44)	0 (0)	

Note. Unless otherwise stated, data are presented as patient numbers with percentages shown in parentheses. The histological information was gathered from specimens obtained via core needle biopsy before chemotherapy. ^a^ Tumor size represented the maximal tumor diameter measured on DCE MR images. Data are presented as means ± standard errors. HER2 human epidermal growth factor receptor 2.

**Table 5:** Breast cancer elasticity values on pretreatment MRI according to the response to neoadjuvant systemic therapy (*n* = 24).

Variables	All (*n* = 24)	Non-Pathological Complete Response (*n* = 18)	Pathological Complete Response (*n* = 6)	*p*-Value ^a^
Mean elasticity value (kPa)	10.26 ± 6.84	12.20 ± 6.71	4.45 ±2.81	<0.001
Minimum elasticity value (kPa)	7.01 ± 4.76	8.11 ± 4.86	3.72 ± 2.55	0.047
Maximum elasticity value (kPa)	14.02 ± 10.75	16.97 ±10.79	5.19 ± 3.22	0.016

Note. Data are presented as means ± standard errors. ^a^ *p*-values represent comparisons between the pathological complete response and non-pathological complete response groups.

## Data Availability

The data presented in this study are available on request from the corresponding author due to ethical reasons.
